# Analysis of the Opportunities, Benefits and Risks Associated with the Use of Recycled Materials in Flexible Aircraft Pavements

**DOI:** 10.3390/ma18133036

**Published:** 2025-06-26

**Authors:** Sean Jamieson, Luke Verstraten, Greg White

**Affiliations:** School of Science, Technology and Engineering, University of the Sunshine Coast, Sippy Downs, QLD 4556, Australia; sjamieson@usc.edu.au (S.J.); lverstra@usc.edu.au (L.V.)

**Keywords:** airport, flexible pavement, life cycle assessment, sustainability, recycling, waste materials, EPDs

## Abstract

International waste policy promotes the reduction and re-use of waste materials, and in some cases, specifically calls for the use of recycled materials in pavements. In countries like Australia, most of the aircraft pavement network is constructed of flexible pavements. Consequently, understanding the opportunities for recycled materials in flexible aircraft pavements is paramount to increasing the technology uptake. This paper reviews opportunities for the incorporation of recycled materials in flexible airport pavement construction, specifically, their application to particle substitution in unbound and asphaltic layers, use in stabilization treatments, and use as a bitumen modifier. Additionally, environmental product declarations are reviewed to provide a range of typical environmental costs for each recycled material when considering material processing for incorporation into flexible pavements. These materials are compared to virgin material environmental costs to determine which recycled materials provide the highest environmental benefit potential. It was concluded that particle replacement in unbound layers with waste materials had a similar environmental cost to using virgin materials. However, the requirement to dispose of waste material to the landfill can be significantly reduced. For asphaltic layers, recycled asphalt pavement as an asphalt mixture replacement, fly ash as a hydrated lime replacement, and waste plastic and crumbed rubber as a virgin polymer replacement all are effective in reducing the environmental cost. To further increase the technology uptake, a risk-based approach for the implementation of waste materials in airport flexible pavements is recommended, which considers performance testing, the depth of the pavement layer, and the pavement functional area.

## 1. Introduction

Flexible aircraft pavements primarily are comprised of granular materials, usually with a bituminous surface layer, over the subgrade, and are constructed as airport runways, taxiways, parking aprons, and shoulders [1,2,3]. They are termed ‘flexible’ because they are intended to deform vertically under load and then rebound to their original shape when the load is removed [1]. Although the design, construction, and maintenance of flexible aircraft pavements is similar to that of roads, the heavier aircraft loads, increased tire pressures, and aircraft engine fragility to loose stones, known as foreign object debris (FOD), result in increased performance requirements and tighter construction tolerances [4]. Consequently, when new flexible pavement technologies are introduced, such as incorporation of recycled materials, the uptake for aircraft pavements is generally slower than that of road pavements [5,6].

International policies for waste management currently promote the reduction and re-use of waste materials [7,8,9] and, in some cases, call specifically for the incorporation of waste materials in the construction of pavements [9]. Consequently, understanding the recycled material application to flexible aircraft pavement construction and quantifying its environmental sustainability benefit are significant in achieving international waste policy targets. Several waste materials have been incorporated in pavement layers, such as waste plastics, recycled crushed glass (RCG), industrial slag, fly ash, crumbed rubber, recycled asphalt pavement (RAP), and recycled concrete aggregate (RCA) [10]. However, each technology has a different level of market maturity [11], meaning that not all materials are used to the optimal content in flexible pavement applications. Furthermore, the application of recycled materials in flexible aircraft pavements is limited due to the risk-averse nature of the airport industry [5].

To optimize the use of waste materials in aircraft pavements, Jamieson, White and Verstraten [6] developed and recommended a set of principles for the use of recycled materials in airport pavement construction, focused on a triple bottom line (TBL) sustainability assessment, sorting and processing, recycled material source location, and performance testing. Included in the TBL assessment was a life cycle assessment (LCA) for environmental sustainability quantification, which is standard practice to assess environmental impacts of a material or process by estimating energy inputs and greenhouse gas emissions for a defined system boundary [12]. The environmental cost of specific construction materials is available in documents known as environmental product declarations (EPDs), which generally assess the environmental impact from raw material extraction through to material manufacturing, based on a defined set of product category rules [13,14]. EPDs allow for comparative assessment of pavement layers using only virgin materials to pavement layers using recycled materials. This is important because not all recycled material pavement applications will provide an overall sustainability benefit [6]. Furthermore, if the incorporation of a recycled material results in significant early-life maintenance, there will likely be a sustainability disbenefit [6]. Consequently, in addition to determining the environmental cost of a pavement layer containing recycled materials, a key principle of incorporating the recycled material is confirming that the resultant layer will perform no worse than an equivalent layer without the recycled component.

This research reviewed the opportunities for using waste materials in flexible airport pavements, specifically, their application to particle replacement in unbound and asphaltic layers, use in stabilization treatments, and use as a bitumen modifier. Additionally, EPDs were reviewed to provide typical environmental costs for each recycled material when considering the required material processing prior to incorporating into flexible pavements. These material costs were compared to virgin material costs to determine which recycled materials provide the greatest environmental benefit potential. Finally, a risk-based approach to the implementation of recycled materials in flexible aircraft pavements was recommended to aid in technology uptake, which must consider performance testing, the depth of the pavement layer, and the pavement functional area.

## 2. Background

### 2.1. Flexible Aircraft Pavements

Flexible aircraft pavement structures are designed, constructed, and maintained in a similar manner to other flexible pavement structures, such as roads and highways [15]. However, the difference in traffic loadings means that the performance outcomes and construction tolerances are generally more demanding for aircraft pavements than for road pavements [16,17]. That is because aircraft pavements are subjected to significantly higher tire pressures and wheel loads than road pavements and must not produce loose aggregate or FOD, which can cause catastrophic damage to aircraft engines [1]. Consequently, the adoption of innovations, such as recycled materials in pavement layers, is generally slower than road pavements due to the higher consequence of pavement failures in an airport context.

**Figure 1:**
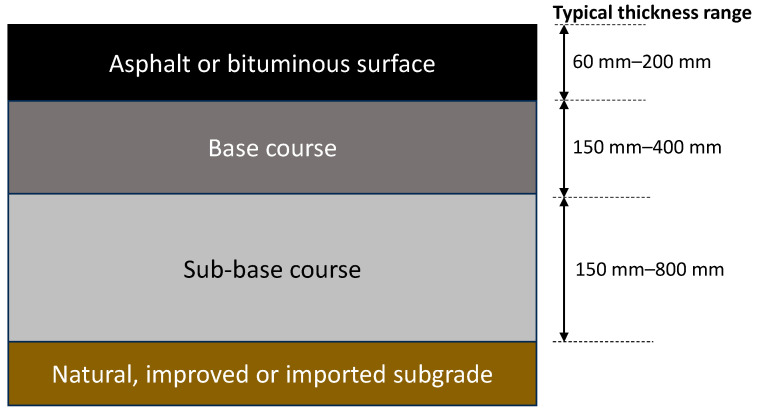
Typical structure of flexible airport pavements.

As shown in Figure 1, flexible aircraft pavements typically comprise a bituminous surfacing over an unbound base and sub-base layer, over a natural, imported, or stabilized subgrade [1,18,19]. Within the pavement structure, each layer spreads the load until the stress is reduced to a level that the subgrade material can accommodate [1]. Consequently, higher quality materials are used for layers closer to the surface, where the stresses are highest, with lower quality materials allowed to be used closer to the subgrade.

The bituminous surfacing can either be an asphalt mixture or sprayed seal [20], with asphalt mixtures preferred for pavements supporting larger aircraft. Bituminous binders within asphalt layers were historically conventional binders. However, in countries like Australia, the use of a polymer modified binder (PMB) is now common [21,22]. The base layer typically comprises a premium-quality, unbound material such as fine crushed rock (FCR), and the sub-base layer can comprise an FCR or a lower-quality aggregate [1,23]. Base layers are sometimes stabilized to increase their modulus, which is achieved with cementitious or foamed bitumen stabilization [2,24,25]. Flexible pavements are typically constructed on areas that are not subject to static loads or hydrocarbons, such as runways, taxiways, and shoulders [1]. In contrast, rigid pavement structures are generally constructed on runway ends and parking aprons. However, it is also common practice for regional airports that support only smaller aircraft to have their entire aircraft pavement network constructed of flexible pavements, including the runway ends and parking aprons [26]. Consequently, due to the multiple layers within a flexible pavement structure and their application to multiple airport areas, there are several opportunities for incorporating recycled materials into different pavement areas and layers.

### 2.2. Waste Materials Commonly Used in Flexible Pavements

Many waste materials can be recycled into flexible aircraft pavement structures. The most commonly used waste materials in pavements include waste plastics, RCG, industrial slag, fly ash, crumbed rubber, RAP, and RCA [10,27,28,29]. Each product has different applications, benefits, and risks. Furthermore, each product will require a different level of processing to be successfully incorporated into a flexible pavement structure, as summarized below.

#### 2.2.1. Waste Plastics

Waste plastics are polymeric materials sourced from industrial or commercial applications [30]. The two most common sources of waste plastic are plastic drink bottles, usually made from polyethylene terephthalate (PET), and single-use plastic bags, usually made from high-density polyethylene (HDPE) [31]. However, there are many other polymers also found in waste plastic. Waste plastics can either be thermoset or thermoplastic polymers. Thermoset polymers undergo a chemical change when heated and cannot be reformed or remelted. Consequently, these are not readily recycled in other applications. In contrast, thermoplastic polymers can be modified by reheating, making them far more suitable for use as recycled product [32]. However, because of the diverse types of polymers, as well as the common presence of contaminants in waste plastic streams, sorting and processing is required before incorporating into flexible pavement structures, which can be energy intensive [33].

#### 2.2.2. Recycled Crushed Glass

RCG is produced from consumer mixed and manufacturing glass waste [34]. However, most RCG is sourced from glass drink bottles [35], which can contain residual sugars and paper contamination [36]. RCG is first sorted by color using optical sensors [37], since the color of the glass is related to the chemical composition and therefore material durability [38]. Because of the waste stream particle size and residual contaminants, RCG also has to be crushed, processed, and cleaned prior to incorporation into pavements, which all require extra energy input [39].

#### 2.2.3. Industrial Slag

Industrial slag is a by-product of the steel and iron making process, and is produced during the separation of the molten steel and iron from impurities in furnaces. The slag occurs as a molten liquid melt and is a complex solution of silicates and oxides that solidifies upon cooling [40]. There are multiple slags produced from steel and iron making, with two common slags used for flexible pavements being ground granulated blast furnace slag (GGBFS) and steel furnace slag (SFS). GGBFS is blast furnace slag from iron manufacturing that has been further processed through grounding and granulation [41]. GGBFS consists of calcium oxide, silica, and alumina, and the pozzolanic properties make it a supplementary cementitious material [42]. SFS is produced from the steel manufacturing process [43] and has a similar appearance to natural aggregates. However, SFS has increased abrasion resistance, higher crushing strength, and increased density [44].

#### 2.2.4. Fly Ash

Fly ash is a coal combustion by-product produced from coal-fired power stations [29]. It is a fine material that has been used in pavements since the early 1950s [45]. There are multiple grades of fly ashes, which are classified by the amount of silica, alumina, iron oxide, and calcium oxide that they contain [46]. Like GGBFS and cement, fly ash demonstrates pozzolanic properties; however, these properties occur at later stages, with fly ash acting more as a filler in early-life construction [47]. Additionally, smaller fly ash particles demonstrate increased pozzolanic properties compared to larger particles [48]. Consequently, fly ash is often used as either a filler material or supplementary cementitious material in pavements [49].

#### 2.2.5. Crumbed Rubber

Crumbed rubber is sourced from end-of-life car, truck, and civil plant tires [50]. The tires contain natural rubber, synthetic rubber, carbon black, oils and resins, metal reinforcement, nylon and rayon fabric, zinc oxide, and curing agents [51]. Additionally, cars, trucks, and civil plant tires will have different quantities of each of these materials [52]. Due to the high rubber content, re-use of end-of-life tires are favored for applications that require rubber polymers, such as bituminous materials. To produce crumbed rubber, waste tires are deconstructed through shredding to remove the non-rubber components such as steel and nylon from the rubber components [51]. Magnets and aspirators are also used to aid the removal of the non-rubber components [53]. The rubber is then further shredded, granulated, and ground to the desired size, which is typically less than 0.6 mm for bitumen applications [51].

#### 2.2.6. Recycled Asphalt Pavement

RAP is the output of old asphalt pavement surface milling or cold planing, and provides an approximate one-for-one replacement for fresh binder and virgin aggregate in an asphalt mixture [29]. Consequently, it is a high-value recycled material for asphaltic layers. When considering RAP for airport surfacing, the source of RAP can significantly affect the performance of the asphalt layer. For example, White and Jamshidi [54] described RAP sourced from temporary ramps that are commonly constructed between runway resurfacing shifts to be low risk, since these ramps are made of the same asphalt mixture used for the surfacing. The highest risk RAP would be drawn from uncontrolled stockpiles that may contain millings from non-airport surface paving projects. However, with suitable stockpile management, RAP performance can be improved [55], and the material can be less variable than newly quarried crushed rock sources [56].

#### 2.2.7. Recycled Concrete Aggregate

RCA is the by-product from the demolition of buildings and old concrete pavements. Crushing and screening of RCA is usually required because unprocessed RCA is often contaminated with timber, steel, and plastics [57]. The source of the RCA has a significant effect on the durability of the material, with one study determining that RCA sourced from vertical structures, such as buildings, has higher durability and strength than RCA sourced from horizontal structures, such as pavements. This was mainly due to the increased presence of contamination in the case of the latter [58].

RCA is generally composed of 60% to 75% aggregates and 25% to 35% adhered mortar [59]. The adhered mortar often leads to RCA being less dense and more porous than virgin aggregates, and this can cause less fragmentation resistance [60], which must be considered when using as a recycled material in flexible pavement applications.

### 2.3. Sustainability Principles for Incorporating Recycled Materials in Aircraft Pavements

Although there are several waste materials that can be used in flexible pavements, detailed engineering analysis is required to determine if the pavement will perform adequately and if the incorporation of the recycled material provides a net sustainability benefit. To aid in the assessment, Jamieson, White and Verstraten [6] recommended principles for incorporating recycled materials in aircraft pavements, which included a TBL assessment, sorting and processing costs, source location assessment, as well as performance testing of the end product, as discussed below.

**Figure 2:**
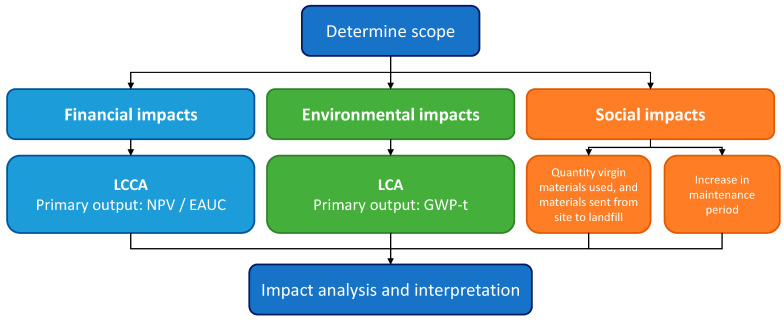
TBL approach for assessing recycled materials in aircraft pavements.

As shown in Figure 2, a TBL assessment determines the financial, environmental, and social costs and allows comparison against a conventional pavement that does not include recycled materials. The financial analysis uses a life cycle cost assessment (LCCA), which considers both the upfront costs, as well as the costs forecast throughout the life of the pavement [61], with the final output being a net present value (NPV) or equivalent annual cost (EUAC). As discussed earlier, the environmental analysis uses an LCA, which is a systematic process that assesses the environmental impact of a product over the entire life cycle of that product, from raw material extraction to material production and manufacturing, use, and end of life treatment [12]. This is similar to LCCA but with environmental cost rates replacing the financial costs.

The most reported output for an LCA is the total global warming potential (GWP-t) [62], which has been established as the principle sustainability metric of interest for pavement-related LCA comparisons [63]. GWP-t is a measure of all atmospheric emissions that contribute to global warming, represented as equivalent kilograms of CO_2_ produced per tonne [64].

The social impacts of the TBL are quantified as the quantity of virgin material consumed, plus the quantity of material sent to the landfill. Although this is also related to environmental benefits, the preservation of natural resources for intergenerational equity is the main focus of social cost when comparing infrastructure that provides the same functional benefit to the community [11]. Additionally, ensuring that the maintenance period is either equal or increased when using recycled materials will reduce social disbenefit, since any major works on airports can significantly affect community access to essential supplies and the ability to travel.

## 3. Recycled Materials Opportunities in Flexible Aircraft Pavements

There are a range of recycled materials that have a demonstrated history in flexible pavement construction, with newer technologies regularly emerging. As discussed above, the most commonly reported materials are waste plastic, RCG, industrial slag, fly ash, crumbed rubber, RAP, and RCA [10]. However, each recycled product will have a different application and will have an optimum rate to ensure both a performance and sustainability benefit. For example, RCA can be used in asphalt mixtures but can provide a net negative environmental sustainability benefit due to the increased binder requirement to account for the porous mortar on the aggregates [65]. However, RCA can be used as an aggregate replacement in unbound pavement layers effectively, with a significant sustainability benefit [29]. Similarly, waste plastics, when used as a virgin aggregate replacement in asphalt mixtures, provide a significant increase in carbon emissions [66], with variable performance benefit [67]. However, when used as a bitumen modifier, there are significant environmental savings and increased performance [31,66].

The primary opportunities of recycled materials in flexible airport pavements include particle replacement in unbound layers, stabilization methods, bitumen modifiers, aggregate replacement in surface courses, and filler replacement in asphalt layers. The maturity of recycled material use in these flexible pavement applications is reflected in their allowable limits in typical road and airfield standards, as shown in Table 1. This table compares specifications from Australian road authorities, from the states of Queensland (QLD), New South Wales (NSW), and Victoria (VIC), against United States (US) Federal Aviation Administration (FAA) airport specifications, and Australian airport practice. For particle replacement applications, maximum limits by percentage of the total mixture are also included. The table demonstrates that larger quantities and types of recycled materials are generally allowed in roads specifications compared to airport specifications, which is due to the more risk-averse nature of the airport industry. Consequently, although there is already some use of recycled materials in flexible airport pavements, there is potential to further increase the volumes and types. However, in all specifications, the performance of the recycled material when used in a flexible pavement application must be confirmed, evidenced by all specifications having some form of performance test. The opportunities and current use of these recycled materials in flexible pavement applications is further discussed below.

### 3.1. Particle Replacement in Unbound Layers

Unbound layers are used for either bases or sub-bases in airport pavements. The purpose of the unbound layers is to spread the load to ensure once it reaches the subgrade, the stresses are weak enough that the subgrade can accommodate [1]. Typically, base layers, which are subjected to higher stresses, will be made of high-quality material, such as FCR, and sub-bases, which are subject to less stress, can use less quality material, such as uncrushed aggregate [83]. This is reflected in current airport thickness design programs that assign a higher modulus to FCR base layers than they do to unbound sub-base layers [2], with a weaker layer modulus requiring increased total pavement thicknesses [84].

FCR is produced by fully crushing sound, un-weathered rock to a pre-defined grading envelope and will generally have a soaked Californian Bearing Ratio (CBR) greater than 100% [83]. In countries like Australia, the importance of having a sound FCR unbound layer is paramount, because thick FCR layers are generally used with only a relatively thin asphalt surfacing [85]. Therefore, the FCR needs to be less susceptible to instability under increased moisture conditions when compared to road pavements [16] and when covered by thicker asphalt courses, as is common in the US and Europe. Consequently, the FCR layers used for aircraft pavements are generally comprised of coarser materials, with lower plasticity than those used for road applications [83].

Furthermore, FCR layers in airports are generally proof rolled to simulate the effects of aircraft-induced loads on pavement layers to identify any deficiencies in the material prior to acceptance of the layer and placement of overlying pavement layers [86]. Consequently, if recycled materials are used as a particle replacement in unbound layers, they should be able to achieve the same proofing regime as virgin FCR layers. Additionally, they should also achieve the same strength properties (modulus or CBR value), because any reduction in modulus value can increase the overall thickness of the pavement, which can negate any sustainability benefit when using the recycled material. The most used materials for particle replacement in flexible pavement unbound layers are RCG, industrial slag, and RCA, as discussed below.

#### 3.1.1. Recycled Crushed Glass

RCG can be used as a fine particle (<4.75 mm) replacement for unbound granular applications and is already allowed in multiple road specifications [29]. For example, Australian road specifications allow 20% to 50% of RCG fines in sub-base layers [68,69] and 10% in base layers [70].

Although the performance of unbound layers containing RCG and those with only virgin material has been shown in the laboratory and by field testing to be similar [36,87], the difference in the allowable percentage reflects the risk appetite of the road authority, with lower pavement layers, that are subjected to less stress, allowed to contain higher contents of recycled material [6].

In fact, other researchers have found that unbound layers can substitute up to 30% by mass of material with RCG fines in sub-base layers and still achieve the required performance when compared to virgin material [88]. However, the use of coarse-grained (>4.75 mm) RCG particles has not been reported to perform as well as fine-grained RCG particles [89]. This is mainly due to the coarse-grained particles exhibiting elongated and flat shapes, leading to high segregation potential during compaction [90]. Consequently, RCG can be used in aircraft pavement unbound-layer applications, but only as a fines material replacement and only if performance is confirmed.

#### 3.1.2. Industrial Slag

Industrial slags produced from blast furnaces have high percentages of fractured faces, which provides good aggregate interlock when used in unbound layers [41]. Although the density of steel slags is typically higher than that of virgin aggregates [91], the mechanical properties important for unbound-layer construction, such as Los Angeles abrasion, sodium sulphate soundness, and flat and elongated particle size, are generally within typical airport unbound base course material limits [40,71]. Consequently, there are many road and airport specifications that allow for large percentages of slag material to be particle replacements for both coarse aggregate and fine aggregate portions of unbound layers [29,71]. Some specifications even allow up to 100% total replacement of virgin aggregate with slag materials [70], as long as the slags do not exhibit unreasonable volumetric expansion, which is an undesirable property of certain steel slags produced using free lime but can be mitigated by appropriate conditioning [44].

#### 3.1.3. Recycled Concrete Aggregate

RCA from vertical and horizontal concrete structures is generally suitable for unbound-layer applications. However, the residual adhered mortar content can result in increased abrasion loss [60] and reduced particle soundness [92] when compared to virgin aggregates. This can result in long-term durability issues for unbound layers. However, when high-quality RCA is used, with appropriate quality control, the abrasion resistance characteristics can be comparable to those for virgin aggregates [93], resulting in RCA base and sub-base layers performing as well as virgin aggregate layers [29]. Consequently, the use of RCA in unbound sub-base and base course layers is common practice globally [59], and RCA has been used in airport pavements as early as the 1960s [94].

### 3.2. Stabilization Treatments

Stabilization is required for flexible aircraft pavements for two different scenarios. The first is to stabilize the in situ subgrade when it does not achieve a CBR of 3% due to the soil’s inability to support the pavement construction [2]. The second reason for stabilization is to bind together granular materials when used for base layers to increase the layer modulus, which reduces the overall pavement thickness required to support the design traffic [1].

Traditionally, subgrades are either chemically stabilized with cement or lime, or mechanically stabilized with shot rocks or cobbles [2]. However, there are recycled materials that can be used for either chemical or mechanical stabilization. For example, fly ash can be used as a chemical stabilization product for expansive clays at 10 to 15% of fly ash by dry weight of soil [95]. Fly ash can improve the unconfined compressive strength, bearing capacity, and resilient modulus of soft soils due to its pozzolanic properties [46]. In fact, most waste materials that have pozzolanic properties can be used for soil stabilization [96], with several examples of industrial slags used to improve soil strength and reduce swelling potential when mixed at various proportions with fly ash [95,96,97]. Regarding mechanical stabilization, materials such as RCA can be used to strengthen subgrades or as a select fill atop low-strength subgrades to allow sub-bases to be constructed above, and is common practice worldwide [59,98].

Sub-base and base layers, when stabilized, are typically done so through cement stabilization or foamed bitumen stabilization [99]. For cement stabilization, an unbound layer is typically treated with 1% to 3% cement content to increase the layer modulus [1]. However, the cement content can be supplemented with supplementary cementitious materials, usually fly ash or GGBFS, or a combination of both [100]. Not only do these waste materials reduce the environmental impact of the pavement construction, but they also allow for an increased working time, which allows for more time to meet specified compaction and rideability requirements [100]. Furthermore, the granular layers that are bound can contain waste material aggregates such as RCA and still perform similarly to layers only containing virgin aggregates [101]. Likewise, foamed bitumen stabilization can take advantage of waste materials. Foamed bitumen stabilization is an expedient construction practice and involves mixing foamed bitumen with a graded aggregate and secondary binder, either in situ or ex situ. The secondary binder is typically lime or cement at 1% to 2% of the mass of the granular material [100]. However, fly ash can also be used as the secondary binder [102], therefore increasing the environmental benefit.

### 3.3. Bitumen Modifiers

Bituminous binders are viscoelastic materials used in the production of asphalt to bind the particles together [103]. Traditionally, airport asphalt production used conventional, unmodified bitumen [21]; however, in the 2000s, several airports reported issues with unmodified bitumen, and it is now common to use modified binders to prevent stripping, horizontal deformation, premature environmental aging, and groove closure [104]. Modified bitumen is also used to satisfy grade bumping requirements when employing the US FAA process for specifying performance-grade asphalts on airports [71].

Modified bitumen generally contains elastomeric or plastomeric polymers at 3% to 7% of the base bitumen mass [105], which enhances the asphalt mixture resistance to fracture and deformation, and increases its durability [3]. The most used polymer modifier is styrene-butadiene-styrene (SBS), which is a thermoplastic elastomer that, when mixed with a bitumen binder, creates a homogeneous polymer network though the bitumen matrix, substantially enhancing the bitumen and asphalt mixture properties [106].

For example, rut depths for wheel tracking tests are significantly decreased when using SBS-modified bitumen compared to non-modified bitumen [107]. However, the use of SBS modifiers considerably increases the GWP-t of asphalt mixtures, with one report demonstrating an 8.6% GWP-t increase for an asphalt mixture when adding 3.5% SBS polymer to the original, conventional base bitumen [108]. Consequently, recycled materials that perform as well as polymer modifiers, but at a fraction of the environmental cost, are highly desirable. Two recycled material products that have demonstrated enhanced bitumen and asphalt mixture performance are waste plastics and crumbed rubber.

#### 3.3.1. Waste Plastics

Like SBS, waste plastics are polymeric materials, and as such, can be incorporated into bitumen to improve the asphalt mixture properties. For example, White [33] demonstrated that a waste plastic-modified bitumen can perform similarly to common Australian elastomeric and plastomeric polymer-modified bitumen in terms of fracture resistance, durability, and deformation resistance. However, in that study, there were reduced fatigue resistance properties for one of the waste plastic modifier products assessed. Additionally, there is already waste plastic-modified bitumen available in New Zealand that satisfies standard polymer-modified bitumen frameworks [109], and it has been used at two international airports [110].

Other studies have also showed enhanced asphalt mixture performance for a wide range of asphalt mixtures using waste plastic modifiers; however, they demonstrated lower fatigue resistance properties at lower temperatures [111]. This highlights the importance of performance testing individual asphalt mixtures. One reason for the variability in performance is that the plastic content in commercial and domestic waste is not homogenous and will include multiple types of polymers such as polypropylene, polyethylene, PET, and HDPE [111].

Some of these plastics, such as PET, have melting points considerably higher than typical binder melting temperatures, meaning that the plastics will stay in their hardened state and not form a homogenous polymer network through the bitumen matrix [31]. Consequently, to ensure the performance of waste plastics as a bitumen modifier, sorting and processing must be conducted [33], which introduces economic and financial costs. However, these costs have been found to be lower than the cost of virgin polymers [66]. Consequently, the use of waste plastics as a bitumen modifier for aircraft pavements can provide an environmental cost benefit without sacrificing performance.

#### 3.3.2. Crumbed Rubber

Crumbed rubber has been used as a bituminous modifier for asphalt mixtures since the 1960s [112], typically at a rate of 20% to 30% by mass of the base bitumen [113]. Crumbed rubber modifiers have been shown to improve asphalt mixture performance properties such as fatigue resistance, durability, rutting resistance, and resilient modulus [52,114]. Crumbed rubber modification is incorporated into an asphalt mixture via field wet blending, terminal wet blending, or dry blending [115]. Wet blending involves fine crumbed rubber being introduced into base bitumen prior to asphalt mixture production, and dry blending is where the crumbed rubber is added directly to the asphalt production plant with the aggregate [113].

The difference between terminal wet blending and field wet blending is the digestion time, during which the crumbed rubber dissolves into the bituminous phase, with terminal blending associated with longer blending times. Dry blending is simpler to implement and less energy intensive; however, when the bitumen is added, the rubber only partially dissolves, resulting in a poorly controlled partial binder blend that does not achieve the full benefits of crumbed rubber modification [116]. In contrast, wet blending leads to a more homogenous and higher-performing mixture, making it more suitable for airport asphalt applications.

As with SBS modifiers, crumbed rubber wet blending will increase financial and environmental production costs when compared to unmodified bitumen [117]. However, the costs are justifiable if the asphalt mixture performs as well as conventional (synthesized) modified mixtures, such as those modified with SBS.

### 3.4. Aggregate Replacement in Surface Courses

Aggregates provide the largest portion of constituent material mass for asphalt mixtures, with around 94% of the constituent materials in airport asphalt mixtures being aggregates [118]. Consequently, because of their criticality to asphalt, premium-quality aggregates are generally used. Typically, aggregates are specified via their consensus properties (angularity, size, and shape), and source properties (abrasion resistance, strength, deleterious material content, and chemical composition) [119], with the latter indicating the long-term aggregate durability [120]. Consequently, to ensure the long-term durability of recycled materials as aggregate replacements in asphalt surface courses, the typical source properties should be achieved. This is especially important for airport asphalt mixtures that rely on stone-on-stone contact to achieve deformation resistance properties, such as stone mastic asphalt [121].

In addition to achieving the source properties, aggregate replacements must have an affinity to bitumen to avoid the risk of asphalt stripping. Furthermore, when using recycled materials as an aggregate replacement, they should not require extra bitumen (as is the case for porous RCA mentioned earlier), which can negate sustainability benefits. Three common waste materials used for asphaltic-wearing coarse-aggregate replacements that have application to flexible aircraft pavements are RCG, SFS, and RAP.

#### 3.4.1. Recycled Crushed Glass

RCG can be used in asphalt mixtures as a fine aggregate replacement, with at least one airport in New Zealand using the waste technology for an aircraft parking apron overlay project in recent years [122]. When using RCG in asphalt mixtures, the optimum bitumen content decreases due to the bitumen not being absorbed into the glass particles at the same level as natural aggregates [37]. This results in less interlock between aggregate particles, reducing the load-bearing capacity of the asphalt mixture [38].

The asphalt mixture performance can be variable when incorporating RCG, with one study demonstrating that up to 15% RCG content had little effect on the asphalt mixture performance [37]. Another study determined that increasing RCG content above 10% of the mixture mass decreased the mixture performance properties to a level not suitable for high-traffic roads [123]. Because of the variable performance, RCG is allowed in heavy-duty road specifications but is generally limited to 2.5% by mass of the total mix if used as a wearing course, and 10% by mass if used as a deeper asphalt layer [75,124]. The low content of RCG in asphalt mixtures and the intensive energy requirement for RCG sorting and cleaning also provides negligible financial and environmental savings when compared to using virgin fine aggregates [39,125]. Consequently, the main benefit for using RCG in airport asphalt mixtures is the reduction of waste glass going to the landfill.

#### 3.4.2. Industrial Slag

Industrial slags, specifically, SFS, have been used throughout New Zealand and Australia as a coarse and fine aggregate material in asphalt and sprayed seal road applications [29], and is also allowed in other international airport applications [71]. Compared to natural aggregates, steel slags have better abrasion resistance, higher crushing strength, and increased density, making them particularly suited to heavily trafficked pavement areas [44].

Performance tests on asphalt mixtures containing steel slags have found reduced rutting potential when tested with a wheel tracking device, and increased fatigue resistance, for both dense graded and stone mastic asphalt mixtures [126]. One study determined that 50% slag (by mixture mass) is optimal for airport stone mastic asphalt applications [127]. Furthermore, due to their affinity for bitumen, steel slags have been found to reduce stripping potential [44]. A potential drawback with steel slags, however, is the requirement to stockpile and wet over a period of months to reduce volumetric expansion due to the free lime content left over from the steel-making process [126], as discussed earlier. Additionally, because of their increased density, transport costs can be increased compared to natural aggregates.

Another consideration is that, due to their thermal properties, mixtures made with slags tend to hold heat longer, allowing for a larger compaction window [128]. Although a large compaction window helps ensure the appropriate asphalt density is achieved, it can also mean a longer time for the asphalt pavement to cool to the ambient temperature, which is generally when aircraft are allowed to commence trafficking. Because asphalt overlays of airport pavement are often performed in reduced working windows, especially for single-runway airports [129], this can potentially have a negative effect on aircraft operations, although only temporary.

#### 3.4.3. Recycled Asphalt

RAP is reclaimed asphalt from an older pavement surface, and in addition to aggregate replacement, it provides an almost one-for-one replacement of bitumen and filler [130]. It has been shown to provide significant environmental and financial benefit, with low performance risk, when compared to other recycled materials [131]. This is mainly due to the replacement of virgin bitumen, which is typically the most expensive constituent material in an asphalt mixture [4].

Asphalt mixtures containing up to 20% RAP have been found to have a negligible performance difference when compared to non-RAP mixtures [132]. However, when the RAP content is above 20%, the quality of the asphalt mixture will be dependent on the quality of the RAP material [133]. Furthermore, the aged binder from the RAP can create an excessively stiff mixture, but this can be mitigated with the use of bitumen rejuvenators that soften the RAP binder [134]. One study demonstrated that up to 40% RAP was suitable for an airport asphalt overlay with a proper rejuvenating agent [135].

RAP is currently allowed in several heavy-duty road and airport asphalt specifications [22], with lower-risk RAP sourced from existing aircraft pavements preferred to un-controlled stockpiles [54]. Consequently, the use of RAP in airport asphalt surfaces provides a high-value and low-risk solution for waste materials in flexible aircraft pavements.

### 3.5. Filler Replacement in Asphalt Layers

Asphalt fillers are defined as the particles that pass the 0.075 mm sieve and can include fines that are naturally present in the combined aggregate portion of the asphalt mixture, and any added fillers [75,136]. The filler content will typically be in the range of 3% to 6% and 8% to 12% of the combined aggregate volume, for dense graded asphalt and stone mastic asphalt, respectively [22,71,137]. Fillers have a significant effect on both mastic stiffness and asphalt mixture performance, with filler shape, texture, particle size distribution, specific surface area, and Rigden voids attributable to the asphalt performance [136]. Typically, mineral fillers are used as added filler and include ground limestone, hydrated lime, and rock dust [81]. However, industrial waste materials also have a significant history of use as added fillers [136]. For other waste materials to be incorporated as an added filler, they must provide suitable mastic and asphalt performance.

Fly ash is a common added filler in both road and airport asphalt specifications [22,81]. Earlier studies have demonstrated that mixtures containing fly ash filler perform similarly or better than those that contain mineral filler [138] and can improve durability and fatigue properties of an asphalt mixture [136]. GGBFS has also been used as an added filler, with mixtures containing GGBFS resulting in a decrease in rutting potential and an increase in stiffness [38]. However, GGBFS does have a significant energy requirement to crush the material when compared to fly ash, which can reduce its sustainability value [139]. Other researchers have also investigated the use of rice husk ash (RHA) as a waste filler. RHA is produced from burning the outer coverings of rice, which makes up 20% of the 500 million tons of paddy rice produced worldwide [140]. The benefits of RHA is that it has a high silica content, pozzolanic properties, and reacts with calcium hydroxide to form cementitious compounds when mixed with water [140]. However RHA has been reported to have variable performance at high temperatures and does not exhibit durability properties as good as other waste products [136]. Consequently, the application to aircraft pavements is expected to be limited.

## 4. Quantifying Environmental Sustainability

**Figure 3:**
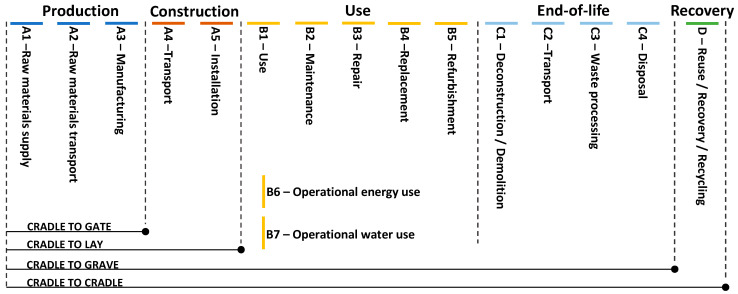
Life-cycled modules for LCA.

As shown earlier in Figure 2, determining the overall sustainability benefit of recycled materials in flexible pavements can be achieved through a TBL approach. Financial impacts are determined through an LCCA, and social impacts are determined by calculating the bulk virgin materials saved and the in situ materials not sent to the landfill. More difficult is determining the environmental cost, which is calculated through an LCA. Although a full LCA includes production, construction, use, end-of-life, and recovery phases [13], as shown in Figure 3, a cradle-to-gate (A1 to A3) assessment is appropriate for initial assessment, since the majority of the carbon cost is experienced in the raw material supply and layer production [141]. This is especially the case if assessing materials that have the same service life and maintenance requirements. However, when comparing materials that have supply terminals situated a considerable distance from the construction site, transport impacts (A4) should be accounted for, which would result in a cradle-to-lay assessment.

Jamshidi and White [142] demonstrated that there can be a wide range in outputs in LCA analysis for a given waste material due to the different properties of unit function, construction technology, service condition, raw material type, mode of transportation, energy cost and productivity, technical skills, and site conditions. Consequently, LCA should be performed for individual project conditions, based on the individual material supply and transport processes. However, in planning and for research purposes, understanding the general range of LCA outputs per recycled materials can provide a suitable estimate of the environmental benefit for each technology, and allows for sensitivity analysis through probabilistic methods such as Monte Carlo simulation [62].

There are multiple sources of LCA data, including EPDs, LCA calculators, Life Cycle Inventory databases, and research articles. There are also significant discrepancies in the data sources, which arise from the scope definition of an LCA study, which establishes the system boundaries for the material under analysis [12]. For example, one LCA calculator states that waste plastics have a zero-carbon cost [143], whereas detailed research by Santos, Pham, Stasinopoulos and Giustozzi [66] demonstrated that waste plastics cost 428 kg CO_2_-eq/t. The difference in the two outputs is that the first analysis assumed that waste plastics were a secondary material that required no further processing. The second analysis included the sorting and processing phases required to enable the material to be used in an asphalt mixture. Sorting and processing is often energy intensive, and it is only required to re-use the material in a pavement application. Consequently, it should be included in LCA [6], meaning that the second analysis is more appropriate.

Of the LCA data sources available, EPDs provide the greatest transparency in determining the environmental effect of a product used in flexible pavements from cradle-to-gate. That is because they provide product-specific detail, are governed by international standards and defined product rules, and are verified by a third party [144]. However, the number of EPDs available for pavement analysis is limited, especially for innovative products such as recycled materials. Nonetheless, with the introduction of government procurement policy that promotes the use of EPDs in project tenders, the quantity of available EPDs is increasing. For example, the Australian government introduced an Environmentally Sustainable Procurement Policy and associated reporting frameworks in July 2024, which requires construction projects that are valued at greater than AUD 7.5 million to report environmental sustainability measures, with one means of compliance being the use of EPDs [145,146]. Since that time, available EPDs in the Australian EPD database have increased significantly, mainly associated with infrastructure construction products [147]. The increase in EPDs reflects the effect procurement policies can have on environmental sustainability and allows more accurate reporting when determining the environmental benefit of recycled materials in flexible pavements.

To enable accurate quantification of the environmental sustainability of waste materials in flexible pavements, Table 2 was developed to describe GWP-t outputs for cradle-to-gate LCA. The values are primarily sourced from existing Australian and New Zealand EPDs. However, for products that had minimal data, international EPDs, LCA calculators, and recent research articles were also used. Where international data was used, caution must be applied if assessing the material in the Australian context. That is because regional and country-specific energy and production requirements will affect GWP-t. For example, when analyzing the asphalt mixture cradle-to-gate environmental cost in the US, Miller, et al. [148] demonstrated that the state and climatic region the asphalt plant was located in had statistical significance for the GWP-t magnitude for A2 and A3, respectively. In that study, the maximum mean GWP-t difference between states was 37.4 kg CO_2_-eq/t for A2 and 4.6 kg CO_2_-eq/t for A3.

**Figure 4:**
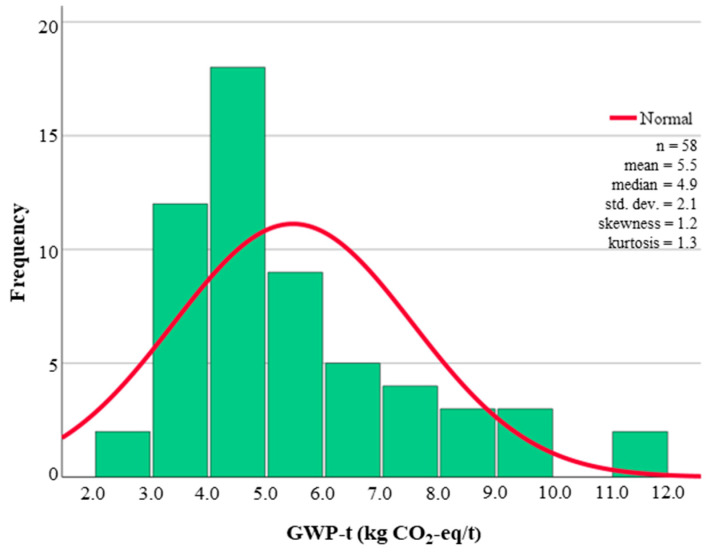
Histogram and normal distribution for virgin coarse and fine aggregates A1 to A3.

**Figure 5:**
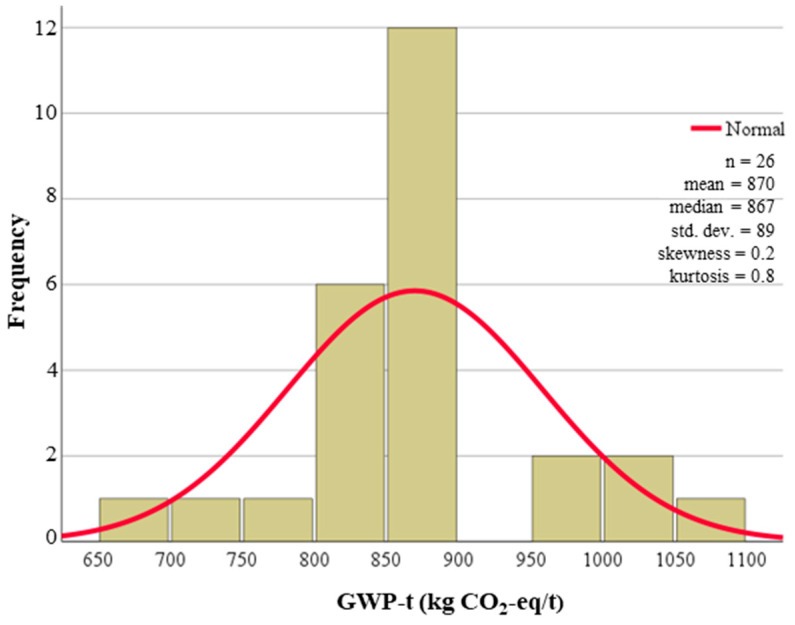
Histogram and normal distribution for cement A1 to A3.

In addition to the range of GWP-t presented in Table 2, there are statistical properties of the sample size (n) and mean value. These values are provided to aid future Monte Carlo analysis of recycled materials in flexible materials, where probability density functions are used as inputs for analysis. Two of the materials investigated had a sample size larger than 10, which allowed for greater statistical analysis with histograms and normal distributions, produced in Figure 4 and Figure 5 for virgin aggregates and cement, respectively. The larger sample size of virgin aggregates and cement was due to the increased market maturity when compared to the emerging markets of recycled material production and supply.

As demonstrated in Table 2, waste materials that can be used for particle replacement in unbound layers and asphaltic layers (steel slag, RCA, and RCG) all have similar ranges of GWP-t values. Consequently, the environmental sustainability benefit will be site specific and likely influenced by the distance from the supply terminal to the project site. However, in all cases, there will be a reduction in material going to the landfill, providing a significant social benefit.

Cement, bituminous binder, hydrated lime, and SBS polymers are shown to be the most environmentally expensive raw materials used in flexible pavements. Therefore, any reduction in these materials by replacing them with recycled materials will provide the greatest environmental benefit. For example, if using the midpoint GWP-t values in Table 1 for a 3% cement treated FCR layer, cradle-to-gate cost would be 32 kg CO_2_-eq/t, assuming a negligible mixture production cost. If replacing cement as the binder with a proven triple blend of supplementary cementitious materials, such as 30% hydrated lime, 40% GGBFS, and 30% fly ash [154], the GWP-t cost reduces to 17 kg CO_2_-eq/t, an almost 50% saving in carbon.

Because asphalt mixtures have several constituent materials, there are more opportunities for environmental savings. To demonstrate this, a conceptual mixture design using only virgin materials was compared to a design that used 20% RAP, a design that replaced hydrated lime with fly ash, a design that replaced SBS polymers with the equivalent quantity of waste plastics, and a design that replaced SBS polymers with 25% crumbed rubber, as shown in Table 3. The conceptual design was based on a typical 14 mm DGA airport mixture using a 5.5% bitumen binder, modified with 4% SBS polymers, and 1.5% hydrated lime as added filler [4]. The quantities of recycled materials were based on the previous literature as discussed earlier, and the environmental cost used midpoints from Table 2. A1 transport costs were excluded from the analysis to allow for an equal comparison of materials. However, because haulage distances generally cost between 0.08 kg CO_2_-eq/t per km to 0.22 kg CO_2_-eq/t per km, dependent on truck size [143], the distance of the material from the supply terminal can significantly affect the final environmental cost. An estimated production cost was also included, which was back-calculated from existing asphalt mixture EPDs [150], with the previous literature demonstrating this value is of a similar magnitude to the raw material supply cost [141].

As shown in Table 3, the environmental savings for each of the waste products in an asphalt mixture is similar, with carbon savings ranging from 10.9% to 13.0% when using midpoint values. Consequently, the distance of the recycled material supply terminal from the asphalt production plant will likely be a significant factor in determining which material has the greatest environmental benefit per pavement project. Additionally, although the fly ash mixture, waste plastic mixture, and crumbed rubber mixture have slightly better savings than the RAP mixture, they are at their maximum content, whereas RAP can be increased well beyond 20% with a detailed mixture design. Furthermore, using RAP will save the largest quantity of material going to the landfill. Consequently, RAP has the greatest potential to provide the largest sustainability benefit. There are also opportunities to combine multiple waste products in asphalt mixtures. For example, using 20% RAP, 1.5% Fly ash, and a crumbed rubber-modified binder could provide up to a 32% environmental saving for A2 to A3. However, the performance of the mixture would need to be proved; otherwise, early-life failure could negate any sustainability benefit due to increased major maintenance. The importance of performance testing of pavement layers using recycled materials is further discussed below.

## 5. Risk-Based Approach to Implementation

**Figure 6:**
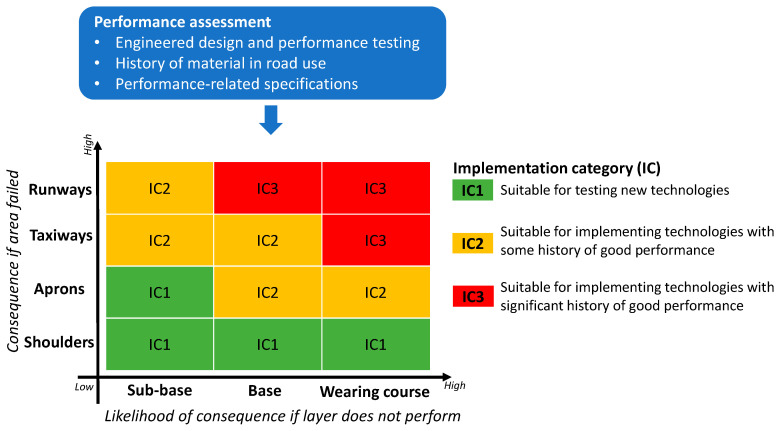
Risk-based approach for using recycled materials in flexible aircraft pavements.

Introducing new technologies into aircraft pavements is difficult due to the generally risk-averse nature of the airport industry [5]. As discussed earlier, this risk-averse nature is the result of the significant consequences of pavement failure when compared to road networks due to aircraft being intolerant of undulations in the pavement surface and their fragility to FOD, which can result in catastrophic damage to aircraft engines [1]. Furthermore, when pavement damage does occur, the resulting maintenance required generally means that portions of aircraft movement areas need to be closed for extended periods of time. For areas like parking aprons, this may not have a large effect on operations. However, when runways are affected, there may be a need to reduce the runway length, resulting in aircraft weight restrictions to satisfy minimum field lengths and, therefore, reduced payload [155]. This will mean a limitation on passengers or supplies to local communities [6], which can reduce airport and airline profits. Consequently, when introducing new technologies such as waste materials into aircraft pavements, a risk-based approach is appropriate. A risk-based approach was developed that considers the location of the pavement, the pavement layer, the performance of the new technology, and the resultant implementation category, with a schematic presented in Figure 6 and discussed in detail below.

The first element of the risk-based approach is to confirm that the pavement material containing the recycled materials can perform adequately. Ideally, performance-related specifications should be used [11], which assess the overall performance of the layer in question when using the recycled materials and confirming it performs as good or better than when using only virgin materials. However, specifications can take significant time to develop, and in countries like Australia, there are limited airport pavement specifications available [16]. Consequently, engineered design and performance testing is required for each specific pavement layer developed with recycled materials.

For example, asphalt mixture surfaces must meet the same requirements for deformation resistance, fatigue resistance, surface texture and skid resistance, and durability as mixtures without recycled materials [3]. Similarly, the unbound layers must meet the normal requirements for soaked strength, proving, and the repeated load modulus [86,156]. Furthermore, supplementary cement bound layers must achieve the normal requirements for unconfined compressive strength or flexural strength [101], and subgrade stabilization should demonstrate an appropriate increase in bearing capacity and a decrease in shrink–swell potential. For performance requirements that are difficult to test in the laboratory, such as age-related durability, experience from road or other pavements should inform the application to aircraft pavements, since age-related durability is largely an environment-related issue and is not specific to aircraft pavements because it is not load related [157].

**Figure 7:**
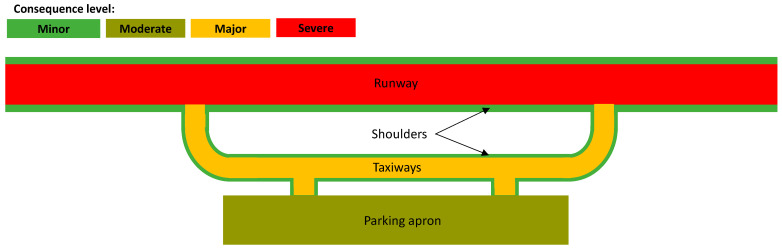
Airport pavement areas and consequence level for unserviceability.

The second element of the risk-based approach is determining the area of the airport that the waste technology will be applied in. Aircraft movement areas generally consist of runways, taxiways, parking aprons, and shoulders [158], as shown in Figure 7.

Runways are used for the take-off and landing of aircraft and therefore have the highest performance requirements [1]. For regional airports that only have one runway, any closure to that runway can significantly affect the ability of the airport to serve communities in facilitating business travel, tourism, and emergency response [159]. Consequently, pavement failure of runways will have great consequence. Taxiways and aprons have less severe consequences if they become unserviceable due to the slower speeds that aircraft operate. However, taxiway serviceability can affect aircraft throughput and the overall aerodrome capacity [160]. Therefore, taxiways will have a higher consequence if they become unserviceable than aprons would. This is especially the case for regional airports that generally have only one taxiway connecting the apron to the runway [26]. Furthermore, aircraft pavement shoulders are usually designed for ground vehicular traffic and irregular aircraft traffic in the case of departure from runways, taxiways, and aprons [2,161]. Accordingly, because they do not service regular aircraft traffic, they present the lowest consequence if becoming unserviceable due to pavement distress.

The third element of the risk-based approach is the depth of the pavement layer that incorporates the waste technology. Because flexible pavements experience less stresses deeper in the pavement [1], using waste material technology at lower layers, such as the sub-base and select fill, will provide a lesser likelihood of consequence should the material not perform. That is, bituminous surface layers will provide the greatest likelihood of consequence if it fails due to the higher stresses experienced at the surface, its direct interaction with aircraft tires, and the potential to create FOD.

The final element of the risk-based approach is determining an implementation category that relates the performance history, pavement location, and pavement layer to a recommendation on where waste material technology can be incorporated in a flexible pavement. The combination of airport area and pavement depth informs what level of performance assessment is appropriate. The higher the implementation category, the more testing, assessment, and historical performance should be demanded for the recycled material proposed to be used.

For example, as shown in Figure 6, shoulders provide a good location for testing new technologies in sub-base, base, and wearing course layers due to the minor consequence should the pavement layer fail. In contrast, runway wearing courses should only use waste technologies that have a significant history of good performance or mature airport specifications. For example, if an asphalt mixture including RAP has been performance tested, and due to the significant history of good performance on roads, there is no reason why it should not be considered for a runway surface. This approach is already reflected in current aircraft pavement practice, where airport-specific material specifications are often required for asphalt mixture and cementitious concrete mixtures used as pavement surfaces, whereas sub-base layers are often permitted to be supplied to road specifications.

## 6. Conclusions

Based on a detailed review of recycled materials in flexible aircraft pavements and current EPDs, it was concluded that particle replacement in unbound layers with recycled materials had a similar environmental cost to using virgin materials for unbound layers. However, the requirement for sending material to the landfill can be significantly reduced, providing a significant social benefit. For stabilization methods, replacement of lime and cement with supplementary cementitious materials can also provide environmental benefits due to the high environmental cost of these binders. For asphaltic layers, using 20% RAP as an asphalt mixture replacement, 1.5% fly ash as a hydrated lime replacement, and waste plastic and crumbed rubber as a virgin polymer modifier replacement are all effective in reducing the environmental cost, with similar cost reductions. However, the use of RAP decreases the landfill requirement the most, and the content of RAP can readily be increased, therefore providing the highest potential sustainability benefit. Finally, implementation categories for using recycled materials in flexible airport pavements were developed based on a risk-based approach that considers performance testing, the pavement layer, and the pavement functional area. Placing waste materials in the non-surface layers of runway shoulders, taxiway, and apron pavements represents the lowest possible risk and should not be resisted for established waste material re-use in the future.

## Figures and Tables

**Table 1:** Presence of recycled materials in typical road and airfield specifications.

Application	QLD	NSW	VIC	FAA Airport	Australian Airport	Reference
Particle replacement in unbound layers (including % of total mixture)	**Base** RCA—100% RAP—15% **Sub-base** RCA—100% RAP—45% RCG—20%	RCA—100% RAP—40% RCG—10% Slag—100%	**Base** RCA—10% RAP—10% RCG—10% Slag—10% **Sub-base** RCA—100% RAP—50% RCG—50% Slag—50%	**Base ** RCA—100% for lower base layers only Slag 100% **Sub-base ** RCA—100% RAP—limit not specified	No existing specification	[2,68,69,70,71]
Stabilization treatments	Fly ash GGBFS	Fly ash GGBFS Powdered glass	Fly ash GGBFS	Fly ash GGBFS	No existing specification	[68,71,72,73]
Bitumen modifiers	Crumbed rubber Proprietary binders including recycled materials	Crumbed rubber	Waste plastic Crumbed rubber	Performance graded, allowing virgin and recycled materials	Proprietary binders including recycled materials.	[22,71,74,75,76,77]
Aggregate replacement in surface courses (including % of total mixture)	**Surface course** RAP—15% RCG—2.5% **Non-surface course** RAP—15% for PMB RAP—40% for conventional binder RCG—10%	**Surface course** RAP—15% for PMB RAP—20% for conventional binder RCG—2.5% **Non-surface course** RAP—10% for PMB RAP—50% for conventional binder RCG—10%	**Surface course** RAP 10%, only with conventional binder mixes. RCG 5% **Non-surface course** RAP 40% RCG as natural sand replacement	**Surface course ** Slag—limit not specified RAP—30%, on shoulders only **Non-surface course ** Slag—limit not specified RAP—30%	**Surface course** Project specific, but up to 8% low-risk RAP without mixture design **Non-surface course** Project specific, but default 20%	[22,71,75,78,79,80]
Filler replacement in asphalt layers	Fly ash	Fly ash	Fly ash GGBFS	Fly ash GGBFS	Fly ash	[22,71,72,79,81,82]

**Table 2:** GWP-t based on the recent literature for flexible pavement materials (A1 to A3).

Material	Application	GWP-t (kg CO_2_-eq/t)	Statistical Properties	Reference
Coarse and fine aggregates	Virgin particles	2.4–11.7	number = 58 mean = 5.5	[143,149,150]
Natural sands	Virgin particles	2.9–5.4	number = 7 mean = 3.8	[143,150]
Steel slag	Particle replacement in unbound layers Aggregate replacement in asphalt and sprayed seals	2.0–7.0	number = 2 mean = 4.5	[144,149]
RCG	Particle replacement in unbound layers Aggregate replacement in asphalt mixtures	3.1–14.9	number = 4 mean = 9.9	[39,143,149,150]
RCA	Particle replacement in unbound layers	3.7–16.0	number = 10 mean = 5.9	[149,150]
RAP	Asphalt mixture replacement	0.0–0.8	number = 2 mean = 0.4	[143,149]
Cement	Stabilization treatment	677–1060	number = 24 mean = 870	[143,149,150]
Fly ash	Stabilization treatment Filler replacement	0–13.7	number = 2 * mean = 6.9	[149,151]
GGBFS	Stabilization Filler replacement	149–177	number = 3 mean = 163	[144,149,150]
Hydrated Lime	Stabilization treatment Filler	1060–1110	number = 4 mean = 1085	[150]
Bitumen binder	Asphalt mixtures	425–605	number = 2 mean = 515	[143,149]
SBS polymers	Binder modifier	3869–5380	number = 3 mean = 4424	[143,152,153]
Waste plastic	Binder modifier	237–428	number = 2 mean = 332	[66,149]
Crumbed rubber	Binder modifier	285–454	number = 4 mean = 365	[51,53,143,149]

* Considered a no-waste product in several Australian EPDs, with a zero environmental allocation factor.

**Table 3:** Environmental cost using conceptual asphalt mixture designs (A2–A3).

Constituent Materials	Virgin Mixture Design		RAP (20% of the Mixture)		Fly Ash (1.5% of the Mixture)		Waste Plastic (4% of Base Bitumen)		Crumbed Rubber (25% of Base Bitumen)	
	% by mass	kg CO_2_-eq/t	% by mass	kg CO_2_-eq/t	% by mass	kg CO_2_-eq/t	% bymass	kg CO_2_-eq/t	% bymass	kg CO_2_-eq/t
Particles	93	6.6	74.4	5.3	93	6.6	93	6.6	93	6.6
Hydrated lime	1.5	14.1	1.2	11.3	0	0	1.5	14.1	1.5	14.1
Bitumen binder	5.2	26.8	4.2	21.4	5.2	26.8	5.2	26.8	5.2	26.8
SBS polymer	0.3	14.1	0.2	11.3	0.3	14.1	0	0	0	0
RAP	0	0	20	0.1	0	0	0	0	0	0
Fly ash	0	0	0	0	0	6.9	0	0	0	0
Waste plastic	0	0	0	0	0	0	0.3	1.0	0	0
Crumbed rubber	0	0	0	0	0	0	0	0	1.4	5.1
A2 total	-	61.5	-	49.3	-	47.6	-	48.4	-	47.0
Production (A3)	-	50	-	50	-	50	-	50	-	50
A2 to A3 total	-	111.5	-	99.3	-	97.6	-	98.4	-	97.0

All percentages are by mass of either the asphalt mixture or the bituminous binder, as noted.

## Data Availability

No new data were created or analyzed in this study.

## References

[1] AAA (2017). Airport Practice Note 12—Airfield Pavement Essentials.

[2] (2021). Airport Pavement Design and Evaluation.

[3] White G. (2018). State of the art: Asphalt for airport pavement surfacing. Int. J. Pavement Res. Technol..

[4] Jamieson S., White G. Review of the performance and cost of asphalt mixture options for runway surfacing. Proceedings of the 8th Eurasphalt & Eurobitume Congress.

[5] White G. (2016). Challenges for Australian Flexible Airport Pavements. Aust. Geomech..

[6] Jamieson S., White G., Verstraten L. (2024). Principles for Incorporating Recycled Materials into Airport Pavement Construction for More Sustainable Airport Pavements. Sustainability.

[7] European Commision (2023). Identifying Member States at Risk of not Meeting the 2025 Preparing for Re-Use and Recycling Target for Municple Waste, the 2025 Recycling Target for Packaging Waste and the 2035 Municapl Waste Landiflling Reduction Target.

[8] US Environmental Protection Agency (2020). The New National Recycling Goal.

[9] Australian Government (2019). National Waste Policy Action Plan.

[10] ARRB (2022). Best Practice Expert Advice on the Use of Recycled Materials in Road and Rail Infrastructure: Part A Technical Review and Assessment.

[11] Groves S. (2023). Standards to Facilitate the Use of Recycled Material in Road Construction.

[12] (2006). Enviornmental Management, Life Cycle Assessment, Principles and Framework.

[13] (2021). Sustainability of Construction Works. Environmental Product Declarations. Core Rules for the Product Cateogry of Construction Products.

[14] (2006). Environmental Labels and Declarations—Type III Environmental Declarations—Principles and Procedures.

[15] White G., Jamieson S. Flexible Airport Pavements as Perpetual Pavement Structures. Proceedings of the Perpetual Pavement Conference.

[16] Jamieson S., White G. Standardised Specifications for Flexible Airport Pavements. Proceedings of the 19th AfPA International Flexible Pavement Conference.

[17] Rodway B., Wardle L.J. Layered Elastic Design of Heavy Duty and Industrial Pavements. Proceedings of the AAPA Pavements Industry Conference.

[18] Chai G., Bell P., McNabb K., Wardle L., Oh E. Comparison of Flexible Airfield Pavement Designs Using FAARFIELD v1.42 and APSDS 5.0. Proceedings of the 12th International Conference on Road & Airfield Pavement Technology.

[19] Wardle L., Rodway B. Advanced Design of Flexible Aircraft Pavements. Proceedings of the 24th ARRB Conference.

[20] White G. Comparison of bituminous surface options for regional airport runway pavements. Proceedings of the 8th International Conference Bituminous Mixtures and Pavements.

[21] Emery S.J. Bituminous surfacings for pavements on Australian airports. Proceedings of the 24th Australia Airports Association Convention.

[22] (2023). Performance-Related Aiport Asphalt Specification—Version 2.1.

[23] Rodway B. Flexible aircraft pavement surfacing—Australian practice. Proceedings of the Eighth International Conference on Maintenance and Rehabilitation of Pavements.

[24] Weir T., White G. Laboratory comparison of in-situ, ex-situ and laboratory produced foamed bitumen stabilised base. Proceedings of the RILEM INternational Symposium on Bituminous Materials.

[25] Priastiwi Y.A., Sukamta, Hidayat A., Hafidz M., Widyandika R., Wiguna E. (2023). Study optimization cement content of cement treated base (CTB) using compressive strength parameter. IOP Conf. Ser. Earth Environ. Sci..

[26] White G., Farelly J., Jamieson S. Estimating the Value and Cost of Australian Aircraft Pavement Assets. Proceedings of the ASCE International Airfield and Highway Pavements Conference 2021.

[27] Salehi S., Arashpour M., Kodikara J., Guppy R. (2021). Sustainable pavement construction: A systematic literature review of enviornmental and economic analysis of recycled materials. J. Clean. Prod..

[28] Gkyrtis K., Pomoni M. (2024). An Overview of the Recyclability of Alternative Materials for Building Surface Courses at Pavement Structures. Buildings.

[29] (2022). Guide to Pavement Technology Part 4E: Recylced Materials.

[30] ARRB (2022). Factsheet—Recycled Plastics.

[31] White G., Reid G. Recycled Waste Plastic for Extending and Modifying Apshlat Binders. Proceedings of the 8th Symposium on Pavement Surface Characteristics: SURF2018.

[32] Kibria M.G., Masuk N.I., Safayet R., Nguyen H.Q., Mourshed M. (2023). Plastic Waste: Challenges and Opportunities to Mitigate Pollution and Effective Management. Int. J. Environ. Res..

[33] White G. Evaluating Recycled Waste Plastic Modification and Extension of Bituminous Binder for Asphalt. Proceedings of the Eighteenth Annual International Conference on Pavement Engineering, Asphalt Technology and Infrastructure.

[34] ARRB (2022). Factsheet—Recycled Crushed Glass.

[35] Harrison E., Berenjian A., Seifan M. (2020). Recycling of waste glass as aggregate in cement-based materials. Environ. Sci. Ecotechnology.

[36] Fulton B. Use of Recycled Glass in Pavement Aggregate. Proceedings of the 23rd ARRB Conference—Research partnering with Practitioners.

[37] Jamshidi A., Kurumisawa K., Nawa T., Igarashi T. (2016). Performance of pavements incorporating waste glass: The current state of the art. Renew. Sustain. Energy Rev..

[38] Jamshidi A., White G. (2020). Evaluation of performance and Challenges of Use of Waste Materials in Pavement Construction: A Critical review. Appl. Sci..

[39] Tushar Q., Salehi S., Santos J., Zhang G., Bhuiyan M.A., Arashpour M., Giustozzi F. (2023). Application of recycled crushed glass in road pavements and pipeline bedding: An integrated environmental evaluation using LCA. Sci. Total Environ..

[40] Chesner W.H., Collins R.J., Mackay M.H. (1998). FHWA-RD-97-148—User Guidelines for Waste and By-Product Materials in Pavement Construction.

[41] ASA (2002). A Guide to the Use of Iron and Steel Slag in Roads.

[42] ARRB (2022). Factsheet—Ground Granulated Blast Furnace Slag.

[43] ASA (2014). Quick Reference Guide 3—Steel Furnace Slag.

[44] VicRoads (2011). Technical Note 09—Steel Furance Slag Aggregate.

[45] ACAA (2003). Fly Ash Facts for Highway Engineers.

[46] Zimar Z., Robert D., Zhou A., Giustozzi F., Setunge S., Kodikara J. (2022). Application of coal fly ash in pavement subgrade stabilisation: A review. J. Environ. Manag..

[47] Li G., Zhou C., Ahmad W., Usanova K.I., Karelina M., Mohamed A.M., Khallaf R. (2022). Fly Ash Application as Supplementary Cementitious Material: A Review. Materials.

[48] Hamada H.M., Abed F., Al-Sadoon Z.A., Alashkar A. (2024). Enhancing pozzolanic activity of fly ash via dry and wet milling: A comparative study for sustainable construction material enhancement. J. CO2 Util..

[49] Mistry R., Roy T.K. (2016). Effect of using fly ash as alternative filler in hot mix asphalt. Perspect. Sci..

[50] ARRB (2022). Factsheet—Crumb Rubber.

[51] TSA (2024). Life Cycle Assessment of End-of-Life Tyres Version 6.1.

[52] Alfayesz S.A., Suleiman A.R., Nehdi M.L. (2020). Recycling Tire Rubber in Asphatl Pavements: State of the Art. Sustainability.

[53] Tushar Q., Santos J., Zhang G., Bhuiyan M.A., Giustozzi F. (2022). Recycling waste vehicle tyres into crumb rubber and the transition to renewable energy sources: A comprehensive life cycle assessment. J. Environ. Manag..

[54] White G., Jamshidi A. Extending the Use of RAP in Airport Asphalt Resurfacing. Proceedings of the RILEM International Symposium on Bituminous Materials.

[55] Gao J., Yao Y., Song L., Xu J., Yang J. (2022). Determining the maximum permissible content of recycled asphalt pavement stockpile in plant hot-mix recycled asphalt mixtures considering homogeneity: A case study in China. Case Stud. Constr. Mater..

[56] White G. (2019). Quantifying the impact of reclaimed asphalt pavement on airport asphalt surfaces. Constr. Build. Mater..

[57] ARRB (2022). Factsheet—Crushed Concrete and Brick.

[58] Ardalan N., Wilson D.J., Larkin T.J. (2020). Analyzing the Application of Different Sources of Recycled Concrete Aggregate for Road Construction. Transp. Res. Rec..

[59] Fanijo E.O., Kolawole J.T., Babafemi A.J., Liu J. (2023). A comprehensive review on the use of recycled concrete aggregate for pavement construction: Properties, performance and sustainability. Clean. Mater..

[60] Pasandin A.R., Perez I. (2014). Mechanical propertis of hot-mix asphalt made with recycled concrete aggregates coated with bitumen emulsion. Constr. Build. Mater..

[61] Buttlar W., Haslag J.H. (2022). A Review of LCCA Literature: Key Factors and Interpretations.

[62] Medina T., Calmon J.L., Vieira D., Bravo A., Vieira T. (2023). Life Cycle Assessment of Road Pavements That Incorporate Waste Reuse: A Systematic Review and Guidelines Proposal. Sustainability.

[63] Hayde C., Walker J., Buckingham-Jones T. Whole-Of-Life Sustainability Assessment of Heavy-Duty Pavement Options for Major Road Infrastructure Projects. Proceedings of the ASCP2023—7th Concrete Pavements Conference.

[64] Van Dam T., Taylor P. (2009). Building Sustainable Pavements with Concrete—Briefing Document.

[65] Pasandin A.R., Perez I. Stripping Potenital of Half-Warm Mix Asphalt made with Recycled Concrete Aggregates. Proceedings of the 7th Eurasphalt and Eurobitume Congress.

[66] Santos J., Pham A., Stasinopoulos P., Giustozzi F. (2021). Recycling waste plastics in roads: A life-cycle assessment study using primary data. Sci. Total Environ..

[67] Lee S.-Y., Kim K.-W., Yun Y.-m., Minh Le T.H. (2024). Evaluation of eco-friendly asphalt mixtures incorporating waste plastic aggregates and additives: Magnesium, fly ash, and steel slag. Case Stud. Constr. Mater..

[68] VicRoads (2017). RC 500.01 Code of Practice Registration of Crushed Rock Mixes.

[69] TMR (2022). MRTS05 Unbound Pavements.

[70] TfNSW (2020). QA Specification 3051—Granular Pavement Base and Subbase Materials.

[71] FAA (2018). AC 150/5370-10H—Standard Specifications for Construction of Airports.

[72] TRMS (2018). QA Specification 3211 Cements, Binders and Fillers.

[73] TMR (2022). MRTS08 Plant-Mixed Heavily Bound (Cemented) Pavements.

[74] VicRoads (2023). RC 500.01 Code of Practice Registration of Bituminous Mix Designs.

[75] TMR (2023). Asphalt Pavements, MRTS 30.

[76] TfNSW (2023). QA Specification 3252—Polymer Modified Binder for Pavements.

[77] (2023). Standard Specification for Performance-Graded Asphalt Binder.

[78] VicRoads (2023). Technical Note TN 107—Use of Recycled Materials in Road Pavements.

[79] VicRoads (2021). Section 407—Dense Graded Asphalt.

[80] TfNSW (2021). QA Specification R116—Heavy Duty Dense Graded Asphalt.

[81] TMR (2017). MRTS103 Fillers for Asphalt.

[82] (2024). Standard Specification for Mineral Filler for Apshalt Mixtures.

[83] Emery S. (2015). Pavements and surfacings on WA airports. Aust. Geomech. J..

[84] Nassiri S., Shafiee M.H., Khan M.R.H., Bayat A. (2015). Evaluation of variation in unbound layers’ moduli and its effect on pavement design. Int. J. Geotech. Eng..

[85] Anstee H., White G. Closing the Fine Crushed Rock Proof Rolling Gap in Airport Pavement Construction. Proceedings of the International Airfield & Highway Pavment Conference.

[86] Anstee H., White G. Defining the Gap Associated with Compacting and Proving Granular Layers and Fills for Airport Pavement Construction. Proceedings of the International Conference on Transportation and Development.

[87] Doukani A., Bekki H., Hariche L. (2024). Grain Size Correction of Pavement Unbound Granular Material Using Recycled Glass Aggregate. KSCE J. Civ. Eng..

[88] Ali M.M.Y., Arulrajah A., Disfani M.M., Piratheepan J. (2011). Suitability of Using Recycled Glass-Crushed Rock Blends for Pavement Subbase Applications. Geo-Front..

[89] Mohajerani A., Vajna J., Cheung T.H.H., Kurmus H., Arulrajah A., Horpibulsuk S. (2017). Practical recycling applications of crushed waste glass in construction materials: A review. Constr. Build. Mater..

[90] Disfani M.M., Arulrajah A., Bo M.W., Hankour R. (2011). Recycled crushed glass in road work applications. Waste Manag..

[91] Kumar P., Shukla S. (2023). Utilization of steel slag waste as construction material: A review. Mater. Today Proc..

[92] Pereira P.M., Vieira C.S. (2022). A Literature Review on the Use of Recycled Construction and Demolition Materials in Unbound Pavement Applications. Sustainability.

[93] Cancino P.F., Ali S.A., Zaman M., Vellingiri A., Hobson K.R., Rojas-Pochyla J. Assessment of Durability and Strength of Recycled concrete Aggregate (CRA) for Use in Pavement Base Construction. Proceedings of the International Airfield and Highway Pavements Conference.

[94] Hironka M.C., Brownie R.B., Wu G.Y. (1981). Recycling of Portand Cement Concrete Airport Pavements.

[95] Yildirim I.Z., Prezzi M. (2022). Subgrade stabilisation mixtures with EAF steel slag: An experimental study followed by field implementation. Int. J. Pavement Eng..

[96] Tanyıldızı M., Uz V.E., Gökalp İ. (2023). Utilization of waste materials in the stabilization of expansive pavement subgrade: An extensive review. Constr. Build. Mater..

[97] Sajid M.H., Rahman M.W., Roy E., Debnath A., Ridoy T. Stabilization of Soil with Fly Ash and GGBS for Subgrade and Backfill Material. Proceedings of the International Conference on Planning, Architecture & Civil Engineering.

[98] Maduabuchukwu Nwakaire C., Poh Yap S., Chuen Onn C., Wah Yuen C., Adebayo Ibrahim H. (2020). Utilisation of recycled concrete aggregates for sustainable highway pavement applications; a review. Constr. Build. Mater..

[99] Stephenson G., Shah A. Pavement Stabilisation as a Sustainable Pavement Recycling Treatment Option for Brisbane City Council. Proceedings of the 3rd Australian Pavement Recycling and Stabilisation Conference.

[100] Austroads (2019). Guide to Pavement Technology Part 4D: Stabilised Materials.

[101] Crucho J.M.L., Picado-Santos L.G.d., Neves J.M.C.d. (2024). Assessment of the durability of cement-bound granular mixtures using reclaimed concrete aggregate and coconut fiber. Constr. Build. Mater..

[102] Weir T., White G., Espinosa R. (2021). Review of the design, characterisation and production of foamed bitumen stabilised base courses for pavement construction. Aust. J. Civ. Eng..

[103] Austroads (2014). Austroads Guide to Pavement Technology Part 4B: Asphalt.

[104] White G. Binder for Airport Asphalt Surfacing. Proceedings of the 17th AAPA International Flexible Pavements Conference.

[105] Austroads (2008). Austroads Guide to Pavement Technology Part 4F: Bituminous Binders.

[106] Zhang Q., Wang T., Fan W., Ying Y., Wu Y. (2014). Evaluation of the properties of bitumen modified by SBS copolymers with different styrene-butadiene structure. J. Appl. Polym. Sci..

[107] White G., Embleton K. Validation of a New Generation Bitumen for Airport Asphalt. Proceedings of the 17th AAPA International Felxible Pavements Conference.

[108] Mukherjee A. (2021). Update to the Life Cycle Assessment for Asphalt Mixtures in Support of the Emerald Eco Label Environmental Product Declaration Program.

[109] Hogan F. Sustainable Products and Processes. https://www.fultonhogan.com/wp-content/uploads/2020/09/Sustainable-Product-PortfolioSept2020.pdf.

[110] Hogan F. Fulton Hogan’s Plastiphalt—The Circular Economy in Action. https://www.fultonhogan.com/fulton-hogans-plastiphalt-the-circular-economy-in-action/.

[111] You L., Long Z., You Z., Ge D., Yang X., Xu F., Hashemi M., Diab A. (2022). Review of recycling waste plastics in asphalt paving materials. J. Traffic Transp. Eng. Engl. Ed..

[112] Kidd A., White G., Stephenson G. Estimating the cost and Benefit of low Dosage Crumb Rubber Modification of Dense Graded Asphalt Mixtures for Longer Lasting Road surfaces. Proceedings of the 19th AfPA International Flexible Pavements Conference.

[113] White G., Kidd A., Shadforth T. (2023). Effect of low dosage crumbed rubber on the mechanical properties of a dense graded asphalt mixture. Road Mater. Pavement Des..

[114] Jamshidi A., Kurumisawa K., Nawa T., Jize M., White G. (2017). Performance of pavements incorporating industrial byproducts: A state-of-the-art study. J. Clean. Prod..

[115] Picado-Santos L.G., Capitao S.D., Neves J.M.C. (2020). Crumb rubber asphalt mixtures: A literature review. Constr. Build. Mater..

[116] Denneman E., Lee J., Raymond C., Choi Y., Yean Khoo K., Dias M. (2015). P31 and P32 Optimising the Use of Crumb Rubber Modified Bitumen in Seals and Asphalt.

[117] Riekstins A., Haritonovs V., Straupe V. (2022). Economic and environmental analysis of crumb rubber modified asphalt. Constr. Build. Mater..

[118] White G. Developing a Performance Specification for Airport Asphalt. Proceedings of the17th AAPA International Flexible Pavements Conference.

[119] Bessa I.S., Castelo Branco V.T.F., Soares J.B. (2012). Evaluation of different digital image processing software for aggregates and hot mix asphalt characterizations. Constr. Build. Mater..

[120] White G. Towards a Performance-Based Airport Asphalt Specification. Proceedings of the International Conference on Highway Pavements and Airfield Technology.

[121] Jamieson S., White G. Developing a Performance-Based Specification for Stone Mastic Apshalt as an Ungrooved Runway Surface. Proceedings of the International Airfield and Highway Pavements Conference 2019.

[122] Downer Downer Case Study: Award Winning Airport Project. https://www.downergroup.co.nz/award-winning-airport-project.

[123] Kalampokis S., Kalama D., Kesikidou F., Stefanidou M., Manthos E. (2023). Assessment of Waste Glass Incorporation in Asphalt Concrete for Surface Layer Construction. Materials.

[124] NSW Government (2021). Heavy Duty Dense Graded Asphalt, Specification R116.

[125] White G., Sorensen L., Jamshidi A. Evaluation of glass as a sand replacement in asphalt. Proceedings of the 18th AAPA International Flexible Pavements Conference.

[126] Pasetto M., Baliello A., Giacomello G., Pasquini E. (2023). The Use of Steel Slags in Asphalt Pavements: A State-of-the-Art Review. Sustainability.

[127] Peng Y., Le W., Jian Z. Research on the Application of Steel Slag AR-SMA-13 Asphalt Mixture in Airport Pavement. Proceedings of the International Conference on Road and Airfield Pavement Technology 2023.

[128] Benavides D., Rangel R.L., Franci A., Aponte D. (2024). Effect of steel slag on compaction times of asphalt mixtures based on prediction of cooling curves. Constr. Build. Mater..

[129] White G. Design and Construct Contracts for Airport Asphalt Resurfacing. Proceedings of the 5th GeoChina Interantional Conference.

[130] Jamieson S., White G. Quantifying the Financial and Environmental Benefits of Recycled Asphalt Pavement in Asphalt Concrete Mixtures for Surfacing of Perpetual Airport Pavements. Proceedings of the Perpetual Pavement Conference.

[131] Van Den Huevel D., White G. Objective comparison of sustainable asphalt concrete solutions for airport pavement surfacing. Proceedings of the International Conference on Sustainable Infrastructure.

[132] Hajj E.Y., Sebaaly P.E., Kandiah P. (2010). Evaluation of the use of reclaimed asphalt pavement in airfield HMA pavements. J. Transp. Eng..

[133] Austroads (2015). AP-T286-15–Maximising the Re-Use of Reclaimed Asphalt Pavement. Outcomes of Year Two: RAP Mix Design.

[134] Stephenson G., Petho L., Giustozzi F. Use of High RAP Content Asphalt on Old Cleveland Road, Brisbane. Proceedings of the 19th AfPA International Flexible Pavement Conference.

[135] Su K., Hachiya Y., Maekawa R. (2009). Study on recycled asphalt concrete for use in surface course in airport pavement. Resour. Conserv. Recycl..

[136] Chen Y., Xu S., Tebaldi G., Romeo E. (2022). Role of mineral filler in asphalt mixture. Road Mater. Pavement Des..

[137] Blazejowski K. (2011). Stone Mastic Asphalt Theory and Practice.

[138] Al-Suhaibani A., Tons E. (1991). Properties of fly ash extended asphalt concrete mixtures. Transp. Res. Rec..

[139] White G., Jamshidi A. Incorporating environmentally sustainable asphalt surfaces into airport pavements. Proceedings of the 4th International Symposium on Asphalt Pavements & Environment.

[140] Jwaida Z., Al Quraishy Q.A., Almuhanna R.R.A., Dulaimi A., Bernardo L.F., Andrade J.M. (2024). The Use of Waste Fillers in Asphalt Mixtures: A Comprehensive Review. CivilEng.

[141] Ma F., Sha A., Lin R., Huang Y., Wang C. (2016). Greenhouse Gas Emissions from Asphalt Pavement Construction: A Case Study in China. Int. J. Environ. Res. Public Health.

[142] Jamshidi A., White G. Use of recycled materials in pavement construction for environmental sustainability. Proceedings of the Eighteenth Annual International Conference on Pavement Engineering, Asphalt Technology and Infrastructure.

[143] Start2See (2022). AfPA LCA Calculator for Asphalt, Version 1.3.

[144] IES International EPD System. https://www.environdec.com/library.

[145] Australian Government (2024). Environmentally Sustainable Procurement Policy.

[146] Australian Government (2024). ESP Policy Reporting Framework.

[147] EPD-Australasia Continued Growth in EPD Publications. https://epd-australasia.com/2024/12/continued-growth-in-epd-publications/.

[148] Miller L., Ciavola B., Mukherjee A. (2024). EPD Benchmark for Asphalt Mixtures.

[149] ARRB (2022). Best Practice Expert Advice on the Use of Recycled Materials in Road and Rail Infrastructure: Part B Sustainability Impacts Report.

[150] EPD-Australasia Environmental Product Declaration Australasia. https://epd-australasia.com/epd-search/.

[151] EPDdanmark EPD Database. https://www.epddanmark.dk/uk/epd-database/.

[152] AENOR (2024). Environmental Product Declaration—Styrene-Butadiene-Styrene Polymer.

[153] Shacat J., Willis J.R., Ciavola B. (2024). The Carbon Footprint of Asphalt Pavements—A Reference Document for Decarbonization.

[154] ARRB (2022). Factsheet—Fly Ash.

[155] Narcizo R.R., Jorge C., Caetano M. (2020). The Aircraft Choice Based on the Aircraft Take-Off Runway Length Requirement. J. Aerosp. Technol. Manag..

[156] White G. Performance based evaluation of a crushed rock base with non-compliant grading. Proceedings of the International Airfield and Highway Pavements Conference.

[157] Abouelsaad A., White G. Fretting and Ravelling of Asphalt Surfaces for Airport Pavements: A Load or Environmental Distress?. Proceedings of the Nineteenth Annual International Conference on Highways and Airport Pavement Engineering, Asphalt Technology, and Infrastructure.

[158] ICAO (2022). International Standards and Recommended Practices. Aerodromes Annex 14 to the Convention on International Civil Aviation.

[159] AAA (2023). Taking Flight: The Economic and Social Contribution of Australia’s Airports.

[160] Dönmez K., Aydoğan E., Çetek C., Maraş E.E. (2023). The Impact of Taxiway System Development Stages on Runway Capacity and Delay under Demand Volatility. Aerospace.

[161] USA Department of Defence (2001). UFC 3-260-02 Pavement Design for Airfields.

